# Process Evaluation of a Nutrition and Lifestyle Behavior Peer Support Program for Adults with Metabolic Syndrome

**DOI:** 10.3390/ijerph17082641

**Published:** 2020-04-12

**Authors:** Muhammad Daniel Azlan Mahadzir, Kia Fatt Quek, Amutha Ramadas

**Affiliations:** Jeffrey Cheah School of Medicine and Health Sciences, Monash University Malaysia, Jalan Lagoon Selatan, Bandar Sunway 47500, Malaysia; quek.kia.fatt@monash.edu

**Keywords:** metabolic syndrome, peer support intervention, nutrition, lifestyle, feasibility, process evaluation

## Abstract

Metabolic Syndrome (MetS) is a cluster of risk factors that increases the risk for diabetes and cardiovascular diseases. Lifestyle intervention is the gold standard of MetS management and prevention. Despite the growing positive influence of peer support-based interventions on management of various chronic diseases, its potential among adults with MetS has not been elucidated. We describe the development and process evaluation of a nutrition and lifestyle behavior “*PE*e*R SU*pport program for *AD*ults with m*E*tabolic syndrome” (PERSUADE) using a systematic five-step approach—(i) review of evidence; (ii) focus group discussions; (iii) behavioral matrix development; (iv) module development; and (v) feasibility and process evaluation. High program adherence was recorded with 81.3% of participants attending all peer sessions. Participants’ content satisfaction score was high (93.3%) while peer leadership score was satisfactory (70.0%). There were significant reductions in all anthropometric and metabolic parameters assessed post intervention, except for diastolic blood pressure. Significant correlations were found between reductions in body fat and triglyceride, and content satisfaction. Peer leadership was only significantly correlated with reduction in triglyceride. Future studies can explore aspects of module interactivity, use of social media, and other means to stimulate consistent engagement of participants, as well as extending the implementations to other lifestyle-related diseases.

## 1. Introduction

Metabolic syndrome (MetS) represents a clustering of metabolic risk factors, which reflects an underlying insulin resistance and adipose tissue dysfunction [1]. The risk factors include large waist circumference, high blood pressure, high fasting blood glucose levels, and dyslipidemia. The urgency to manage this clustering is due to mounting evidence that linked MetS with an increased risk of type 2 diabetes and cardiovascular diseases in later years [1] especially in low-middle income countries, such as Malaysia. The findings from the latest National Health and Morbidity Survey (NHMS 2016) suggested that the prevalence of MetS risk factors has increased rapidly in recent years [2], while a few studies showed a remarkably high prevalence of MetS clustering among Malaysians [3]. This is partly a result of rapid socio-economic development, which comes at the expense of healthy diet and active lifestyle [4,5].

Literature suggests strong relationships between various lifestyle habits and risk for MetS [6,7]. For example, a Western or unhealthy dietary pattern with limited variety and low quality have been shown to be one of the main nutritional risk factors for MetS [6]. Physical inactivity and longer screen time have been associated with MetS risk [7,8]. In addition to poor nutrition and physical inactivity, meta-analysis of observational studies showed a poor sleeping pattern to increase the risk for MetS in adults [9], where short sleep duration (less than 6 h) and long sleep duration (more than 8 h) both tend to increase the risk. Hence, it is not surprising that lifestyle behaviors tend to be the first line approach towards prevention and management of MetS and interventions likely to focus on these aspects. However, the number of such interventions in Malaysia is limited, where only one community-based intervention focusing on the improvement of physical activity levels among those with MetS has been reported [10].

As chronic conditions such as MetS often persist long term, there is a need to explore more cost-effective and sustainable interventions for long-term self-management. Lifestyle modifications have been shown to be a better option than medications for long-term prevention of metabolic disorders such as diabetes [11]. However, instilling sustainable and effective lifestyle changes among individuals with MetS is challenging, and this issue has been previously discussed by Pritchett and colleagues [12]. Often, long-term management also tends to not be addressed in published guidelines on chronic disease management, which rather focus on generic dietary and lifestyle changes. In addition to this, patients’ motivation and barriers to change are often overlooked and only have been documented in limited scenarios [13,14,15].

On the other hand, an integration of a peer support framework in lifestyle interventions is showing growing evidence to improve the outcome in chronic diseases [16]. Peer support, consisting of non-hierarchical, mutually beneficial relationships with similar others who face the same health problem, has been leveraged in group-based lifestyle interventions evaluated in the general population [17]. The effectiveness of peer support interventions has been previously reported in the case of obesity [18], diabetes and hypertension [19,20], and cancer [21]. Since peer support promotes activation of public members to have the same health goals by promoting collective behavioral changes, the framework is seen to substantiate lifestyle intervention better than a stand-alone lifestyle intervention [16].

Growing prevalence of MetS in Malaysia sparks the need of a wholesome and cost-effective lifestyle intervention. Hence, a community-based nutrition and lifestyle behavior “*PE*e*R SU*pport program for *AD*ults with m*E*tabolic syndrome” (PERSUADE) was developed. Here, we describe the five-step development and process evaluation of PERSUADE.

## 2. Materials and Methods

**Figure 1:**
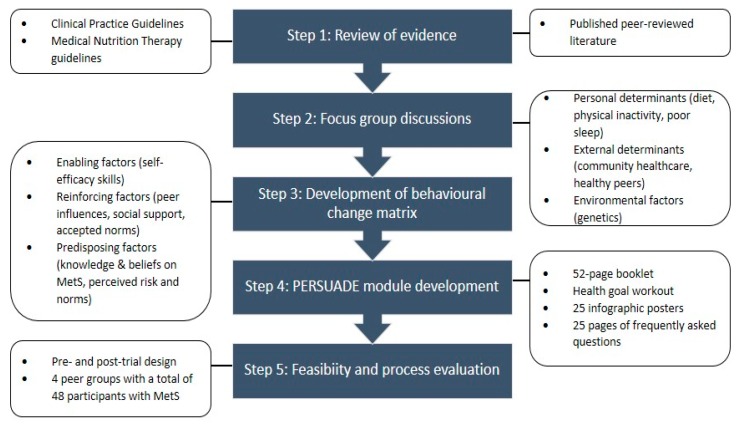
PERSUADE (“*PE*e*R SU*pport program for *AD*ults with m*E*tabolic syndrome”) Peer Module Development Flowchart.

PERSUADE is a peer-based behavioral intervention aimed at Malaysian adults with MetS located in the state of Johor, Malaysia. PERSUADE is designed as a 12-weeks peer support program with the assistance of a peer module that was built using a five-step development process (Figure 1) to ensure that the information that it delivers is evidence-based, appropriate, and community-specific.

### 2.1. Step 1: Review of Evidence

As the first step, we conducted extensive reviews of available established guidelines and literature on lifestyle interventions in MetS among adults. Established guidelines that were reviewed included the National Strategic Plan for Non-communicable Diseases (2010–2014) [22], Malaysian Dietary Guidelines 2010 [23], and clinical practice guidelines for metabolic diseases [24,25,26,27,28], as well as the Malaysian Medical Nutrition Therapy for diabetes, hyperlipidemia, and hypertension [29,30,31]. Specifically, we reviewed objectives related to behavior changes, basic knowledge on MetS components and risk factors, self-management skills, and prevention steps of specific risk factors. In addition, we also researched the guidelines on specific improvement in dietary habits and physical activity.

A literature review of published lifestyle intervention among adults with MetS was conducted to elucidate design approaches and strategies of the interventions. Each intervention was designed closely to the need of the targeted community, and there was no one-size fits all intervention design [32]. Some of the studies however, described the decay of healthy behavior among the participants at post-intervention follow-up. It is worth noting that peer support design has shown to be one of the most resilient approach in terms of healthy behavioral decay and has shown promising evidence in management and prevention of chronic diseases [33].

### 2.2. Step 2: Focus Group Discussions

A qualitative study with a total of 21 purposively sampled respondents was conducted with adults diagnosed with MetS who attended MONASH Medical Precinct. We studied the motivation and barriers of healthy living among adults with MetS via a qualitative study with four focus group discussions. An interview protocol consolidating both responsive interviewing model and Health Belief Model (HBM) [34] was prepared. The focus group discussions were audio recorded and transcribed. Data saturation was achieved in the fourth focus group, and the study was halted.

Thematic analysis was performed on the data. Owing to the explorative nature of the HBM [34] in the qualitative assessment, seven main themes including three motivations and four perceived barriers on healthy nutrition and lifestyle behavior were identified. Motivations identified in the thematic analysis include: (i) weight gain and physical appearances; (ii) personal experience of adverse complications; (iii) good family and social support. The thematic analysis also revealed the following barriers to change: (i) perception that healthcare as a business model; (ii) healthy change is difficult and expensive; (iii) cultural influence on food intake; (iv) inadequate knowledge on MetS. We addressed the factors associated with motivations and barriers elucidated from Step 2 in the development of PERSUADE content in which certain aspects, such as self-sustaining skills, are emphasized more as compared to the others. By doing so, information is designed to be user-friendly, easy to follow, and allowing long-term changes.

### 2.3. Step 3: Development of Behavioral Change Matrix

The PERSUADE behavioral change matrix was then developed using the information gathered from Steps 1 and 2. The matrix served as the backbone of PERSUADE content development, with an aim to combine each specific change objective with specific intervention strategies and delivery methods. Domains of the HBM were used as an adhesive between these three aspects to strengthen the matrix. As the HBM was used as an explanatory framework to study the degree of healthy lifestyle adoption [35], this matrix became a functional template for the module development in Step 4. A part of the behavioral change matrix (focusing on dietary modification) is shown in Table 1.

### 2.4. Step 4: PERSUADE Module Development

PERSUADE aimed to deliver evidence-based, factual, and current health information that will help peers to improve their nutrition and lifestyle behaviors. Hence, we carefully designed each point of information by expanding the matrix with information provided in national guidelines and published literature. Motivations and barriers associated with behavioral change in adults with MetS identified in the qualitative study (Step 2) were incorporated in design of information delivery. As a result, the content of PERSUADE was developed to be community-specific and relevant to the participants.

The PERSUADE Module was designed to be a 12-week peer support program involving adults with MetS. The peers actively interacted with each other with the guidance of the PERSUADE Module on a weekly basis. The lesson plans included a 52-page booklet which comprises of a weekly program schedule, one page of health goal workout, 25 infographic posters, and 25 pages of frequently asked questions given out at the start of the intervention period. Each lesson plan was made available in a simple, understandable form in English and Malay languages. Relevant photographs and illustrations were added to enhance the understanding of the lesson plans. The presentation of information and layout of the posters were kept simple, with session objectives clearly stated in each new section.

The weekly peer module was arranged according to each section to address three main objectives: knowledge of metabolic syndrome, nutrition and dietary target, and healthy lifestyle aims. Each recommendation was also designed to address all related barriers and motivate the participants to change accordingly. A footnote containing various links to websites with related information on MetS, healthy lifestyle, and diet was provided where appropriate.

Sample pages of the module are available as a Supplementary file (S1).

### 2.5. Step 5: Feasibility and Process Evaluation

#### 2.5.1. Feasibility Trial

**Figure 2:**
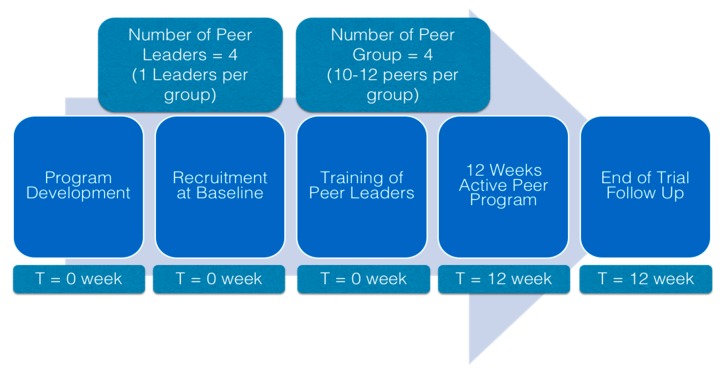
Flowchart of feasibility and process evaluation of PERSUADE.

A pre- and post-trial design was used to assess the feasibility and to conduct the process evaluation of PERSUADE (Figure 2). Recruitment drive was done in two neighborhoods in the state of Johor through health screening events organized by respective neighborhood committees. In order to be eligible, volunteer participants had to be Malaysian and have MetS according to the Harmonized Criteria [36]. In addition, participants had to be willing and able to commit to 12 weeks of intervention. Participants were excluded if they were reported to have cardiovascular diseases, chronic liver or kidney disease, thyroid problems, or advanced cancer. Ethical approval was obtained from Monash University Human Research Ethics Committee, (CF16/56-2016000022) before commencement of the study.

A total of 48 eligible participants were enrolled into the feasibility trial. Forty-four of them were enrolled as peers and divided into four peer groups based on their location. The remaining four participants were trained as peer leaders. A two-day session was planned to inform the leader on the overall peer support framework, essential information on metabolic syndrome, good dietary habits and healthy lifestyle, and behavioral change objectives. Each group was then introduced to their peer leader by the study nutritionist.

Changes in anthropometric measures and metabolic biomarkers were recorded at baseline and 12 weeks post intervention. Height and waist circumference (WC) were measured according to World Health Organization protocol [37] using SECA stadiometer and SECA measuring tape, respectively. Weight and body fat percentage were measured using InBody 120 Body Composition Analyzer. Body Mass Index (BMI) was calculated as weight in kilograms divided by the square of height in meters. Blood pressure (BP) was assessed using OMRON HEM-907XL automated blood pressure monitor. Finger-prick method was used to measure fasting blood glucose (FBG) (B Braun Omnitest 3 Glucometer) as well as triglyceride (TG) and high-density lipoprotein (HDL) (Cardiochek PA Blood Meter).

#### 2.5.2. Process Evaluation

This process evaluation was conducted to assess participants’ adherence to the intervention and satisfaction towards content and peer leadership. The adherence to the intervention was assessed by the peer attendance to each peer session. All participants were required to record their weight before each peer session to ensure validity of the attendance. The program feedback was recorded in a self-administered questionnaire post intervention, assessing the participants’ satisfaction towards the content satisfaction and peer leaders.

Five factors were used to determine the content satisfaction: time and place for peer session, module objective, module content, module structure, and module relevance. Furthermore, peer leadership scores incorporated a few aspects of the program delivery; peer interactions, peer leader readiness, and peer leader knowledge. The responses were recorded using a Likert scale with scores ranging from 1 (strongly disagree) to 5 (strongly agree). Internal consistency of the items was determined using Cronbach Alpha (α), where α > 0.70 denoted good internal consistency. Both content satisfaction and peer leadership components scored excellent in terms of internal consistency with α = 0.921 and 0.898, respectively.

The sum of the responses for each domain (content and peer leadership) reflects the satisfaction of the participants, with higher scores indicating a higher level of satisfaction (Table 2). The sum was then converted into percentage by dividing with maximum score.

#### 2.5.3. Statistical Analysis

Descriptive statistics were used to describe the participants’ demographic characteristics. Normality of all continuous variables was determined using the Shapiro–Wilk test, and data were found not normally distributed. Hence, a Wilcoxon signed-rank test was performed to compare the changes in anthropometric measures and metabolic parameters between baseline and post intervention. Spearman’s rho (*ρ*) was used to assess the correlation between satisfaction towards content and peer leadership and towards changes in anthropometric measures and metabolic parameters. All statistical analyses were performed using IBM SPSS Statistics 25.0, and statistical significance was set at *p* < 0.05.

## 3. Results

### 3.1. Study Participants

Forty-eight adults with MetS with a mean age of 44.17 years (SD = 7.45) were enrolled in the study, of which 52.1% were females. The majority of the participants were Malay (85.4%) followed by Indian (8.3%), and Chinese (6.3%). Almost all (97.7%) participants were working with an average monthly income of MYR3122.92 (SD = MYR1814.98) (approximately USD737.45 (SD = USD428.72)). The participants were locals and had been a resident of the state of Johor for an average of 26.35 years (SD = 17.52 years).

### 3.2. Changes in Anthropometric Measures and Metabolic Parameters

Of the three anthropometric measures assessed in this study, small but significant reductions in median BMI and WC were seen post intervention as compared to baseline (both *p* < 0.001) (Table 3). Median systolic BP was also significantly lower post intervention compared to baseline (*p* = 0.001). Similar reductions were noted in median FBG, TG, and HDL post intervention (all *p* < 0.001).

### 3.3. Process Evaluation

More than 81% of participants attended all peer sessions while 6.3% and 12.5% attended 10 and 11 peer sessions, respectively. Most of the absences were due to work commitments and family issues. There were no dropouts throughout the program. To ensure the validity of each attendance, participants measured their weight during each peer session, which eventually factored into the high program adherences. An individual weight chart was printed and given to each participant to see changes in their weight weekly.

**Figure 3:**
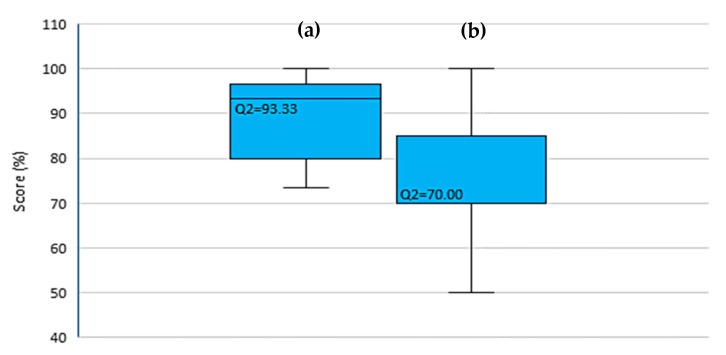
Distribution of study participants according to (**a**) content satisfaction and (**b**) satisfaction towards peer leadership.

All participants satisfied with the content of the module with a median score of 93.33% (IQR = 16.67) (Figure 3). The median score for peer leadership was lower at 70.00% (IQR = 15.00) with two participants disagreeing on each aspect of peer leadership except on peer interaction.

### 3.4. Correlation between Satisfaction and Study Outcomes

There was a small but significant correlation between satisfaction towards intervention content and reduction in body fat content (*ρ* = 0.348, *p* = 0.015) and triglyceride (*ρ* = 0.431, *p* = 0.002). Peer leadership, however, was only correlated with reduction in triglyceride (*ρ* = 0.363, *p* = 0.011). Table 4 presents the correlation analyses.

## 4. Discussion

Interventions that can successfully change diet and lifestyle of individuals with MetS may result in desirable effects by improving their anthropometric and clinical biomarkers. PERSUADE was developed to address the intricate behavioral aspects of Malaysian adults with MetS and their readiness to change in a comprehensive evidence-based, community-specific peer support program.

### 4.1. Outcome of the Feasibility Trial and Process Evaluation

As abdominal obesity is one of the most important components of MetS, many interventions focused on reductions in WC as well as BMI [38]. We noted small but significant changes in WC and BMI post intervention in our study. We have consistently measured body weight of the participants on a weekly basis, and close monitoring of body weight has been shown as one of the factors that could aid with weight loss [39]. Although we found a significant reduction in SBP, such difference was not seen in DBP. In addition to this, the magnitude of change of FBG, TG, and HDL, though significant, was rather small. Past studies have also noted similar issues and suggested a longer period of intervention for us to observe a bigger margin of reductions [40,41].

Satisfaction towards the module content was also observed to be high, signifying a comprehensive five-step approach in module development. The fundamental aspects incorporated in the module development were informed by an extensive review of evidence and guidelines, supplemented by earlier findings from focus group discussions. Hence, the module was tailored to be community-specific. Guenther and colleagues [42] have previously discussed the positive impact of community-specific information in intervention material to increase the participatory rate. In terms of peer support, the study evaluated peer leadership to evaluate interactivity and proactivity of peer leaders in running their individual peer groups. The satisfaction towards peer leadership was relatively low compared to the satisfaction towards PERSUADE’s content. This seems to be a recurring issue with a well-conducted review on peer support, suggesting readiness of peer leaders was often less emphasized, and this factor tends to contribute towards poor delivery and less engagement [43].

We noted a high participatory rate (90%) in PERSUADE. This signifies a high level of motivation in this community to attend and participate in peer-related programs. It has also been shown in multiple peer-based studies that it has been found to be promising in addressing chronic health problems, especially those that are closely related to lifestyle [43]. The design of PERSUADE included an emphasis on empowerment and self-efficacy, making it a person-centered approach. It extended the directive clinical issues with an additional construct that reflects individuals’ values, interests, and problems. This, in return, when harmonized with HBM, reflected the wide variety of social, economic, and community stressors that influence lifestyle-related disease management.

We deduced that several of the following factors could have improved the participation rate in PERSUADE:

#### 4.1.1. Attention to Emotions

People considering peer support often express interest only in information and answers to their questions, not emotional support [43]. Owing to this, PERSUADE was designed as a systematic approach over a period of time. This gives the room for a peer leader to make a gradual evolution of emotional support as trust evolved in the course of instrumental support, such as providing information or help with specific tasks. With the increase trust, peers are giving each other implicit support, such as shared activities; emotional support may be conveyed without explicit discussion of emotions. Implicit support and trust will promote collective changes within the peer group.

#### 4.1.2. Use of Theoretical Model

We used the HBM as the foundation in developing content structure, content, and program delivery. By doing this, we addressed one’s motivation and barrier to change in our content and approach. This increases relevance of this module among the participants and subsequently increases the acceptance rate.

#### 4.1.3. Assistance in Daily Health Management

All peer groups were followed up by their leader and discussed collectively on the planned topics which included MetS as a clustering of risk factors, heart diseases and diabetes, blood sugar levels, blood pressure, healthy eating, and exercise and medication adherences. However, since the aim of PERSUADE is to motivate self-management, this process evaluation noted that an effective creation of individualized behavioral change management plans goes beyond the concept of peer support. The peer discussion appears to have paid little attention to the individualized plans for behavioral change and progress in achieving them that are generally viewed as central to self-management. This is reflected in the understanding of the module content (content satisfaction).

#### 4.1.4. Ongoing Proactive Contact

Our trained peer leaders met the peers every week, and the ongoing process motivated the peer to change and be present for the follow-up. Besides delivering the program, peer leaders were also instilling the sense of collective change and healthy competition among peers in their respective groups.

### 4.2. Limitation

While the program had its strengths, we have identified several limitations or shortcoming that served as lessons learned from the feasibility trial.

Based on verbal feedback received, there were instances where peer leaders unknowingly shared inaccurate information which was noted by peers who have done extensive ‘homework’ in that topic. The satisfaction towards the peer leaders may have been affected if the peers had doubt on the depth of knowledge possessed by their respective leaders. Besides, some peer supporters who tended to be too directive, prescriptive, or bossy in their advice needed to be transferred to other responsibilities. Similar issues were noted in the Peer for Progress project, as they suggested an ongoing supervision for peer leaders in the future construct for peer support [44].

In some instances, extensive focus on the module alone could have discouraged exchange of emotions among peers. At the same time, peer leaders’ communication skills were not emphasized in their training but instead were covered along with several other issues in only one of two days of training sessions. Inclusion of deeper concepts of health psychology could have assisted in these aspects.

PERSUADE was designed to be a community-specific peer-led program. Hence, there may be an issue of generalizability of the program content to other parts of the world. Adaptation of the module to other settings or countries should consider the societal norms and cultural needs of the particular community.

### 4.3. Recommendation for Future Research

Future studies can be designed to be implemented in the real-world setting, incorporating different modes of behavioral change delivery and making a comprehensive integration on emotional and social aspects of program implementation. Moreover, a comparison in terms of cost effectiveness of various modes of interventions can be done.

We would like to highlight several key recommendations for future studies:

1. Emphasis on modifiable nutrition and lifestyle behavior (dietary pattern, dietary timing, diet variety, diet quality, sleeping pattern, smoking cessation, and physical activity programs).
2. Increase the study period from 3 months to a year to promote permanent lifestyle changes.
3. Incorporate technology in monitoring behavioral changes. Improve the rate of acceptance and adherence by including visually possible changes and a tracking system.
4. Improve peer leader training to ensure they are ready and inspiring.
5. Develop a stratified community-based sampling strategy in improve collective changes.
6. Ensure that each peer group is made up of peers with similar socioeconomic background to ensure their timely and relevant discussion throughout the period.
7. Conduct a needs assessment to gauge the societal or cultural needs of a community before adapting PERSUADE.

## 5. Conclusions

Lifestyle changes with the respective risk factor pharmacological management is the common treatment administered to individuals with MetS. Improvement on MetS requires life-long dietary and lifestyle changes supported by a healthy environment. Despite the challenges in delivering lifestyle interventions, the peer support framework has shown to be increasingly evident in promoting lifestyle changes among adults with chronic diseases. In addition to this, peer support programs may address the insufficiency of the current one-off counselling session offered in most public hospital settings for people with MetS-related risk factors.

Process evaluation of PERSUADE shows a high acceptance and adherence rate among participants. Although peer leadership satisfaction can be improved with a more concise training program, the satisfaction towards the module content was high. While the feasibility trial and process evaluation supported the use of peer-based nutrition and lifestyle behavior intervention in this group, the identified limitations, issues, and recommendations can be considered before such program is proposed to a larger population.

## Figures and Tables

**Table 1:** A sample of behavioral change matrix for dietary modification.

Behavioral Objectives	Behavioral Determinants		
	Knowledge	Perceived Benefit	Self-Efficacy Skills
Use Malaysian Food Pyramid and ‘Suku Suku Separuh’ plate to ensure balance, variety, and moderation in amount of consumed food. Use Nutrition Information Panel to understand food contents. Monitor food portions, daily food intake, and food variety.	Describe food classes and food variety using Malaysian Food Pyramid. Describe food portions using ‘Suku Suku Separuh’ representation. Understand what health claims on food products are. Use Nutrition Information Panel to classify healthy foods. Use food diary to track intake.	Ensure balance, variety, and moderation in amount of consumed food is important in balance diet. Know what is in a food product to ensure all ingredients are healthy. Using food records to keep track of calorie intake.	Identify food classes using the food pyramid. Build a healthy plate using ‘Suku Suku Separuh’ concept. Consume a wide variety of foods. Portion food intake to ensure diet is in moderation. Key—“Balance, Colorful, and Moderation”. Identify healthy foods using nutrition information panel. Read health claims in food packaging. Record all food consumed using a food diary.

Note: *Suku suku separuh* (“quarter quarter half”) is a campaign by Malaysian Ministry of Health to encourage adoption of healthy plate.

**Table 2:** Components of the process evaluation.

Components	Scoring Statement	Maximum Score ^a^	Cronbach Alpha (α)
Program adherence	Attendance	12	n/a
Content satisfaction	One hour is enough to learn a topic.	30	0.921
	Place was conducive and comfortable.		
	Module was easy to understand.		
	All information was related to my lifestyle and dietary habits.		
	Each module is systematic and easy to follow.		
	I can use the module to improve my lifestyle and nutrition behaviors.		
Peer leadership	Leader had enough time and interactions to achieve module objectives.	20	0.898
	Leader was prepared to run each peer session.		
	Leader had enough knowledge to run the modules and answer my questions.		
	I can interact with my leader and peers to learn and understand healthy nutrition and lifestyle behaviors.		

^a^ Maximum score shows the maximum possible points obtainable within a component, which is equivalent to 100%.

**Table 3:** Changes in metabolic markers of study participants (N = 48).

	Baseline	Post-Intervention	*P*
	Median (IQR)	Median (IQR)	
Body mass index (kg/m^2^)	25.62 (5.16)	24.99 (4.75)	<0.001 **
Waist circumference (cm)	91.75 (11.40)	91.25 (11.80)	<0.001 **
Body fat (%)	28.75 (7.80)	28.00 (8.40)	0.060
Systolic blood pressure (mmHg)	134.5 (23.00)	128.5 (22.00)	0.001 *
Diastolic blood pressure (mmHg)	80.0 (17.00)	81.0 (15.00)	0.188
Fasting blood glucose (mmol/L)	8.15 (3.00)	7.50 (2.80)	<0.001 **
Triglyceride (mmol/L)	2.71 (0.52)	1.81 (0.51)	<0.001 **
High-density lipoprotein cholesterol (mmol/L)	1.11 (0.48)	1.45 (0.54)	<0.001 **

IQR = interquartile range; * significant at *p* < 0.05; ** significant at *p* < 0.001.

**Table 4:** Correlation between satisfaction towards content and peer leadership, and changes in study outcomes.

Changes in Measures	Content	Peer Leadership
(Baseline Post Intervention)	*ρ*	*ρ*
Body mass index (kg/m^2^)	−0.187	−0.047
Waist circumference (cm)	−0.255	−0.181
Body fat (%)	0.348 *	−0.251
Systolic blood pressure (mmHg)	0.098	0.224
Diastolic blood pressure (mmHg)	0.029	0.034
Fasting blood glucose (mmol/L)	0.026	0.011
Triglyceride (mmol/L)	0.431 *	0.363 *
High-density lipoprotein cholesterol (mmol/L)	0.004	0.073

* significant at *p* < 0.05.

## Supplementary Materials

The following are available online at https://www.mdpi.com/1660-4601/17/8/2641/s1, Figure S1: Sample infographics used in the peer group sessions.
