# Supplementary material for: Process Evaluation of a Nutrition and Lifestyle Behavior Peer Support Program for Adults with Metabolic Syndrome
Source: Int J Environ Res Public Health. 2020 Apr 12;17(8):2641. doi: 10.3390/ijerph17082641 (PMC7215631; doi:10.3390/ijerph17082641)
Supplement: Supplementary file 1 [file ijerph-17-02641-s001.pdf]

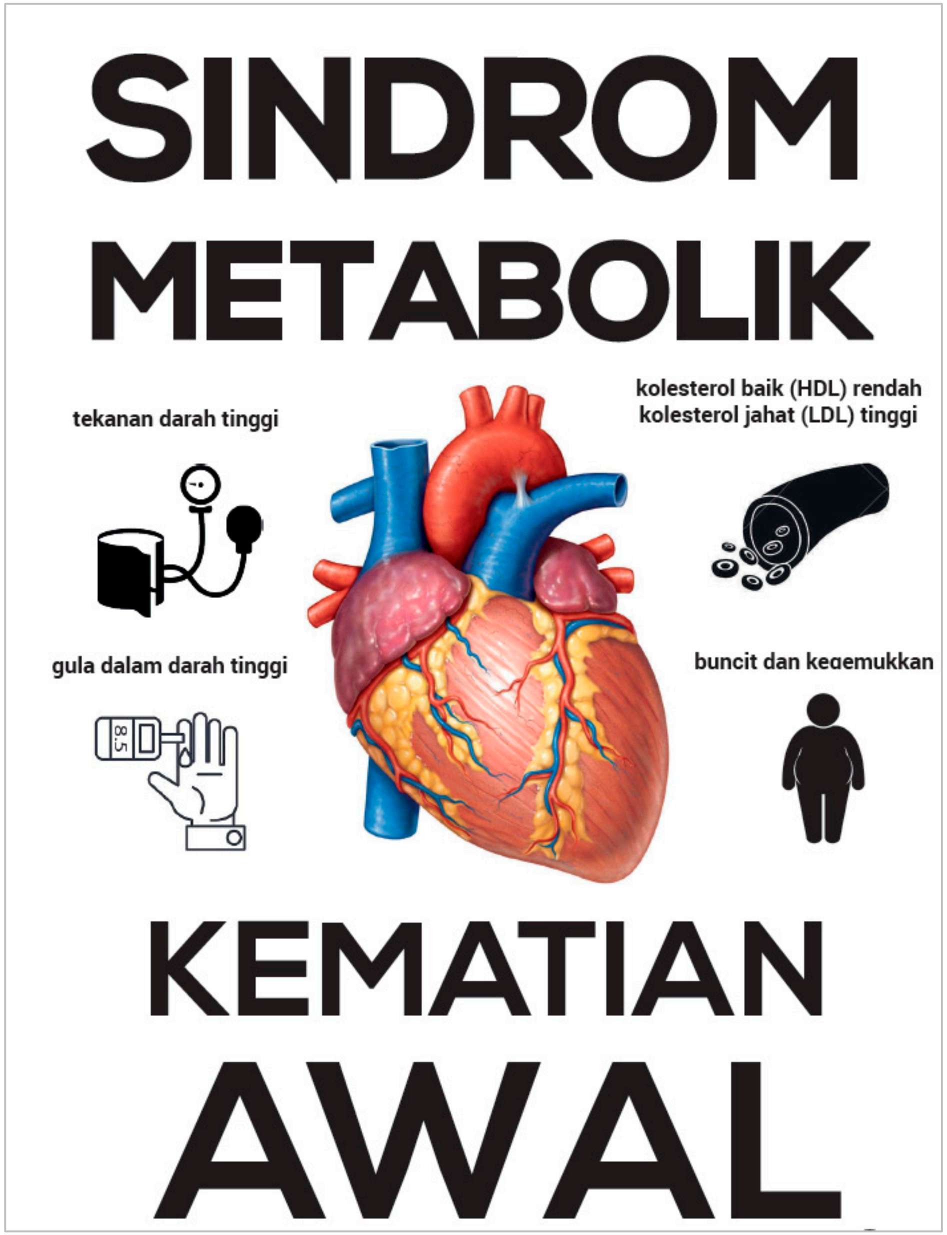


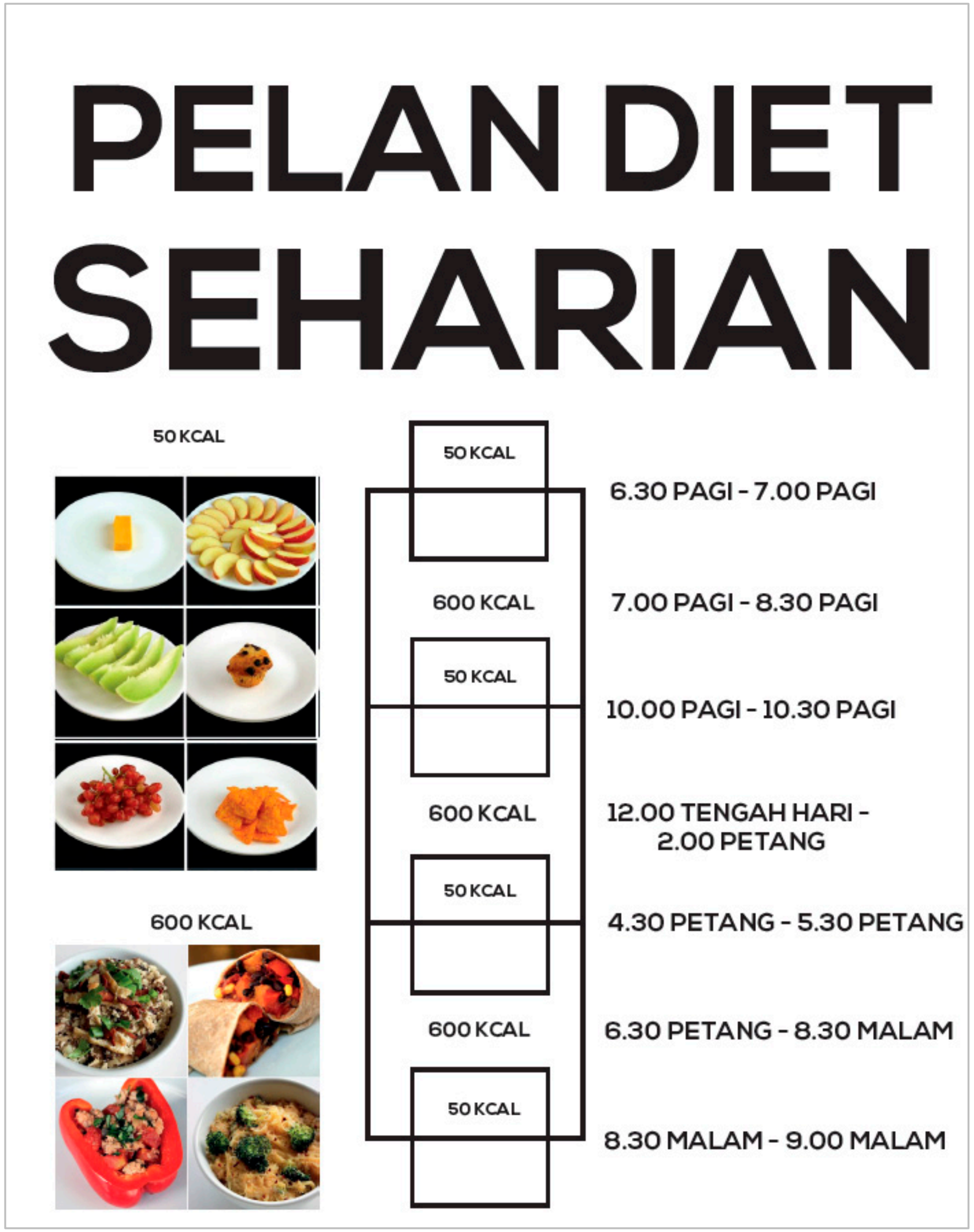


**Figure S1.** Sample pages of booklet given to peer supporters

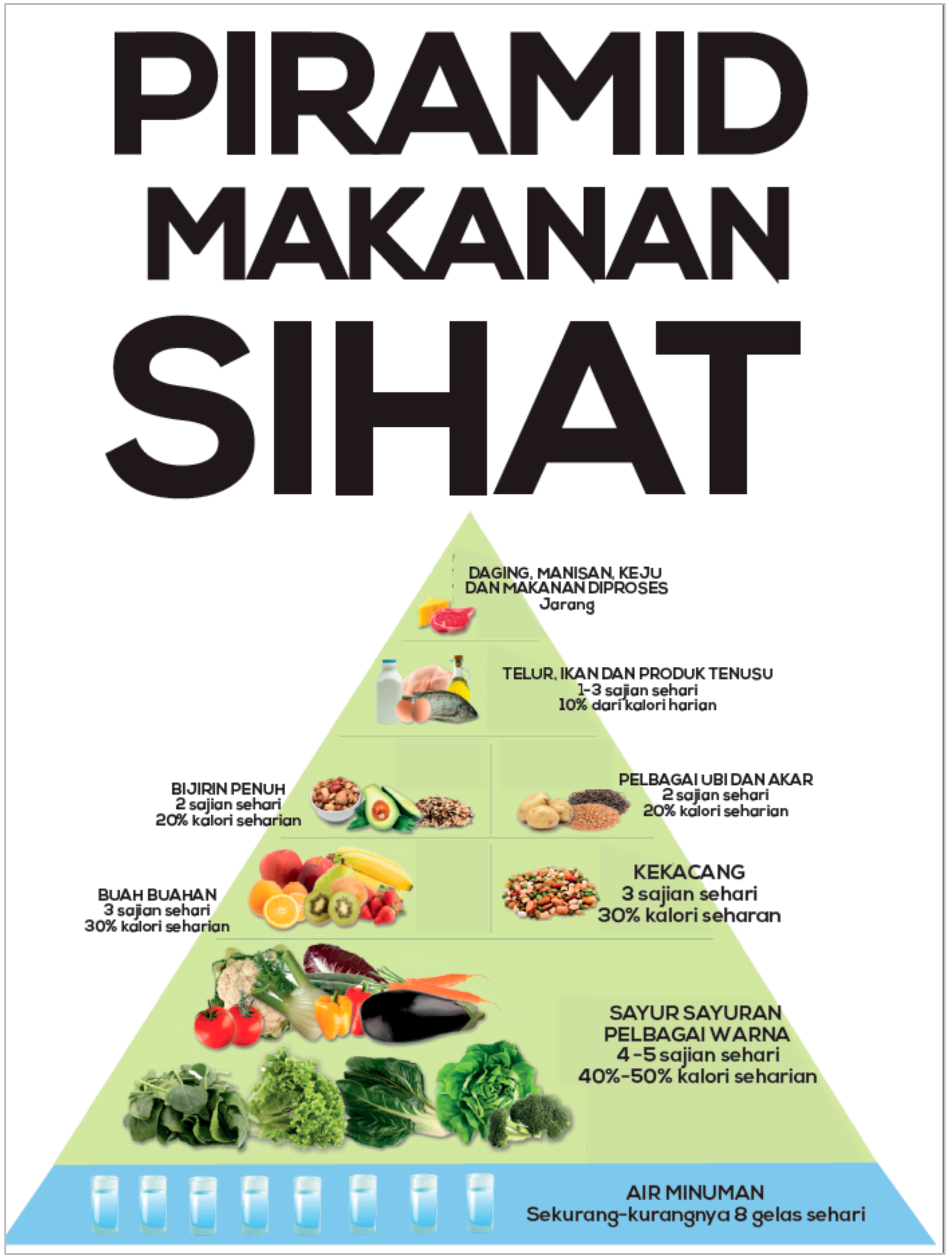


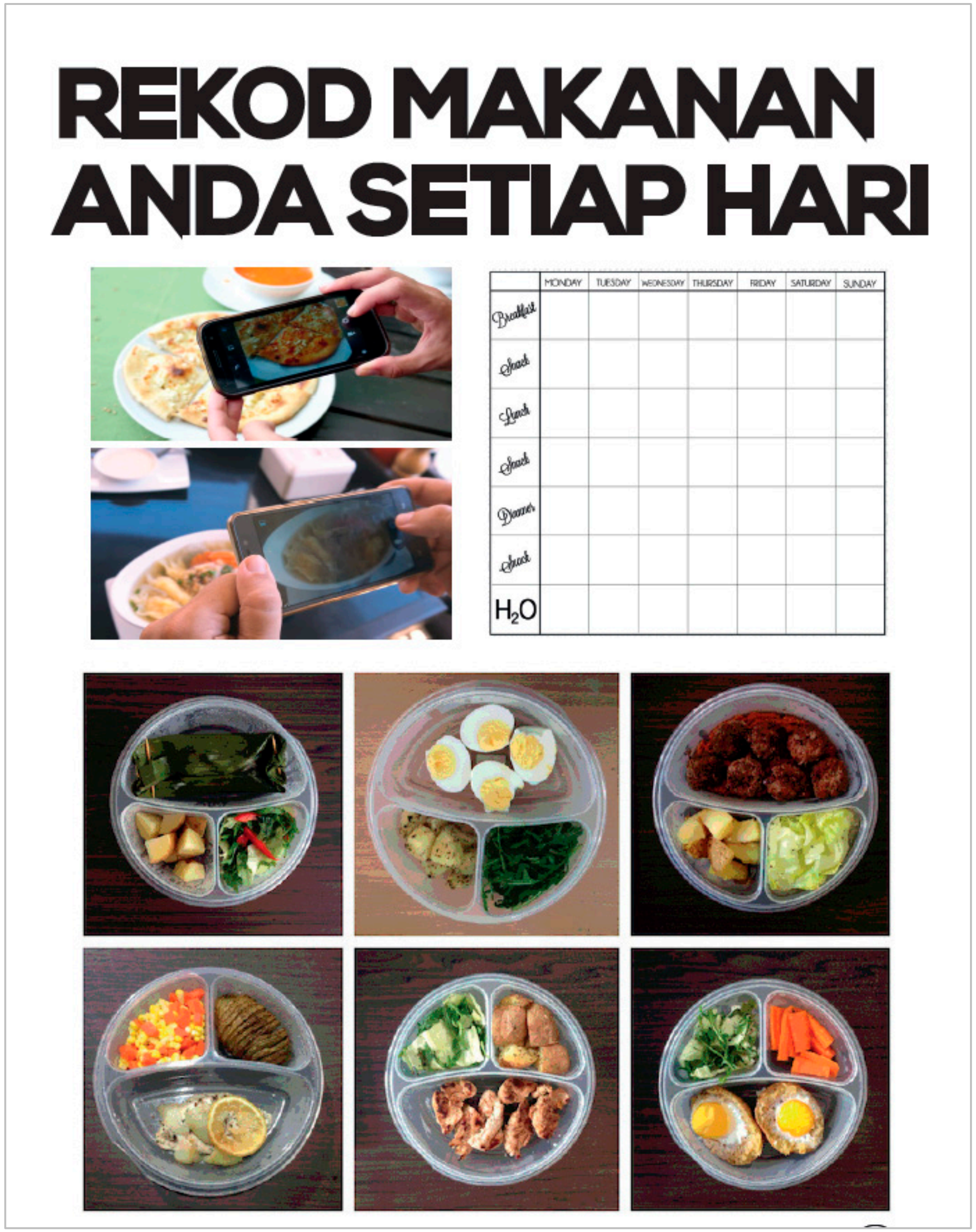


**Figure S1.** Sample pages of booklet given to peer supporters (continued)
